# IFN-γ Signaling to Astrocytes Protects from Autoimmune Mediated Neurological Disability

**DOI:** 10.1371/journal.pone.0042088

**Published:** 2012-07-27

**Authors:** Claudia Hindinger, Cornelia C. Bergmann, David R. Hinton, Timothy W. Phares, Gabriel I. Parra, Shabbir Hussain, Carine Savarin, Roscoe D. Atkinson, Stephen A. Stohlman

**Affiliations:** 1 Department of Neurosciences, Lerner Research Institute, The Cleveland Clinic, Cleveland, Ohio, United States of America; 2 Department of Pathology, Keck School of Medicine, University of Southern California, Los Angeles, California, United States of America; Washington University, United States of America

## Abstract

Demyelination and axonal degeneration are determinants of progressive neurological disability in patients with multiple sclerosis (MS). Cells resident within the central nervous system (CNS) are active participants in development, progression and subsequent control of autoimmune disease; however, their individual contributions are not well understood. Astrocytes, the most abundant CNS cell type, are highly sensitive to environmental cues and are implicated in both detrimental and protective outcomes during autoimmune demyelination. Experimental autoimmune encephalomyelitis (EAE) was induced in transgenic mice expressing signaling defective dominant-negative interferon gamma (IFN-γ) receptors on astrocytes to determine the influence of inflammation on astrocyte activity. Inhibition of IFN-γ signaling to astrocytes did not influence disease incidence, onset, initial progression of symptoms, blood brain barrier (BBB) integrity or the composition of the acute CNS inflammatory response. Nevertheless, increased demyelination at peak acute disease in the absence of IFN-γ signaling to astrocytes correlated with sustained clinical symptoms. Following peak disease, diminished clinical remission, increased mortality and sustained astrocyte activation within the gray matter demonstrate a critical role of IFN-γ signaling to astrocytes in neuroprotection. Diminished disease remission was associated with escalating demyelination, axonal degeneration and sustained inflammation. The CNS infiltrating leukocyte composition was not altered; however, decreased IL-10 and IL-27 correlated with sustained disease. These data indicate that astrocytes play a critical role in limiting CNS autoimmune disease dependent upon a neuroprotective signaling pathway mediated by engagement of IFN-γ receptors.

## Introduction

CNS resident cells are targets of autoimmune mediated damage but also active participants in disease development, progression and control [1], [2]. However, their contributions to neuroprotection and regulation by inflammatory mediators are not well defined. CNS insults, including autoimmune disease, initiate rapid astrocyte activation characterized by cellular hypertrophy and increased intermediate filament glial fibrillary acidic protein (GFAP) expression [3]–[5]. Astrocytes form a physical barrier surrounding areas of inflammation initially limiting bystander tissue damage [6], [7]. However, this barrier subsequently impedes axonal regeneration contributing to sustained disability [3], [5], [8]. Innate and adaptive pro-inflammatory astrocyte functions include production of pro-inflammatory cytokines, reactive oxygen species, chemokines, and matrix metalloproteinases [3]–[5]. By contrast, secretion of anti-inflammatory cytokines and scavengers of reactive oxygen species, as well as inhibition of both microglial activation and tumor necrosis factor (TNF) secretion, all support an astrocyte mediated anti-inflammatory function [3]–[5]. Therefore, astrocyte activation constitutes a ubiquitous, yet heterogeneous response associated with both promoting and inhibiting CNS repair [3]–[5].

**Figure 1:**
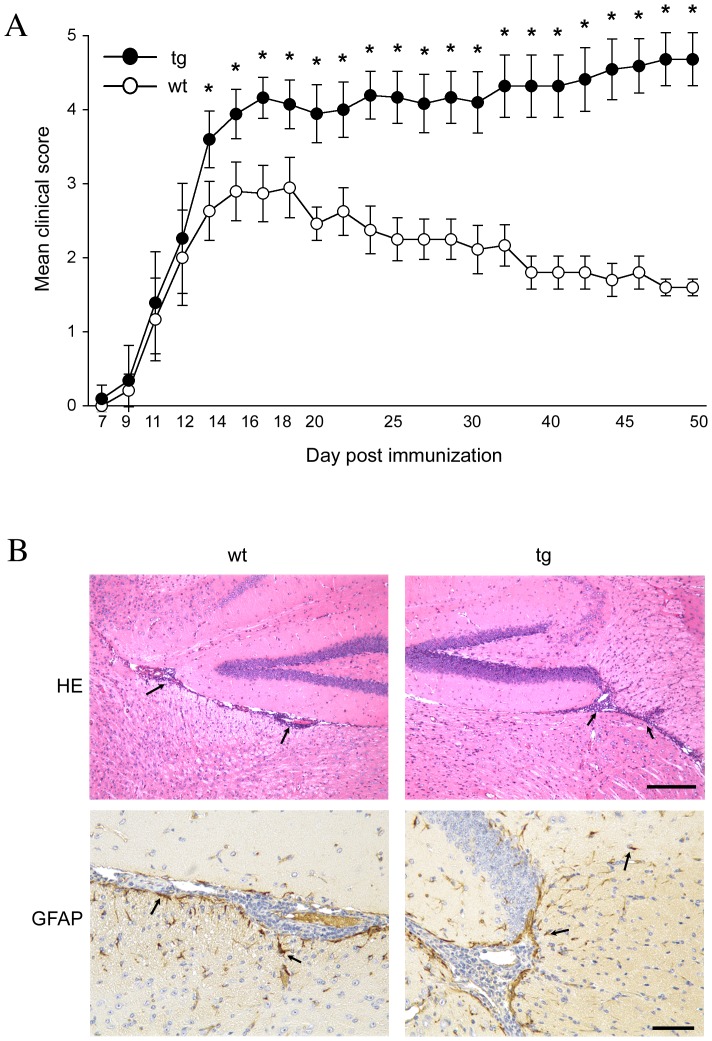
IFN-γ signaling to astrocytes promotes clinical remission. (**A**) Average clinical scores combined from 3 separate experiments with >10 mice per group (GFAPγR1Δ n = 35; wt n = 40). Error bars represent standard deviations. (**B**) Inflammation and astrocyte activation in brain during acute EAE. Inflammation (HE; arrows show perivascular inflammation) and astrocyte activation (GFAP; arrows show activated astrocytes). Paraffin embedded sections from GFAPγR1Δ mice (average clinical score = 3.5) and controls (average clinical score = 2.8) at day 14 p.i. Scale bars: HE = 200 microns; GFAP = 100 microns. *p<0.001.

MS and EAE are both associated with T cells secreting IFN-γ (Th1 cells) and IL-17 (Th17 cells) which play complex, not fully understood roles in disease initiation and progression [2], [9]. In vivo and in vitro evidence indicates that suppression of encephalitogenic T cell proliferation within the CNS during EAE and activation of anti-inflammatory programs are in part mediated via astrocytes [4], [5]. Similar to astrocytes, IFN-γ mediates both pro- and anti-inflammatory functions during autoimmune disease [10]. Early IFN-γ induced effects are pro-inflammatory; IFN-γ facilitates inflammatory cell access, shapes their composition, increases major histocompatibility complex (MHC) expression, contributes to macrophage and microglia activation, and initiates oligodendrocyte death [11]. Similarly, IFN-γ mediated protection during EAE is also multifaceted [12]–[15]. It acts as a negative regulator of neutrophil accumulation, Th17 cell activation, IL-1R signaling, protease secretion, and chemokine activity. It also inhibits pro-inflammatory cytokine secretion via induction of suppressor of cytokine secretion (SOCS) proteins, facilitates T cell apoptosis and protects oligodendrocytes via inducing endoplasmic reticulum (ER) stress responses [10], [11], [16], [17]. This highlights the critical role of a single mediator in both promoting disease but also limiting inflammatory mediated damage required to initiate repair cascades. Based on the gatekeeper functions of astrocytes and the diverse biological effects of IFN-γ, we set out to determine how IFN-γ signaling specifically to astrocytes influences CNS autoimmune disease. The results demonstrate that IFN-γ signaling to astrocytes had no profound effects on initial disease progression, but played an essential protective role during the transition from acute to chronic disease. Clinical remission induced by IFN-γ signaling to astrocytes coincided with reduced demyelination, axonal degeneration, and astrocyte activation. The IFN-γ receptor is expressed ubiquitously; however, these data reveal astrocytes as the primary target and mediator of the well established anti-inflammatory activity of IFN-γ within the CNS.

## Results

### IFN-γ Signaling to Astrocytes Limits Demyelination during Acute EAE

To understand the role of IFN-γ signaling to astrocytes during the pathogenesis of CNS autoimmune disease, EAE was induced in transgenic mice expressing a signaling deficient dominant negative IFN-γ receptor 1 specifically on astrocytes (GFAPγR1Δ mice) [18]. Peripheral activation of self reactive T cells in GFAPγR1Δ mice was similar to wt mice (data not shown). This is consistent with both the CNS restricted transgene expression in the GFAPγR1Δ mice as well as the similar T cell activation following peripheral immunization with a non-self antigen [18]. Following immunization the incidence of disease, initiation of clinical symptoms, and initial symptom progression were unaltered by the inability of astrocytes to respond to IFN-γ (Fig. 1A; Table 1). In addition, neither immunized group exhibited clinical symptoms of atypical EAE associated with the absence of IFN-γ [19]. However, clinical symptoms in GFAPγR1Δ mice began to diverge from wt mice at ∼ day 12 post immunization (p.i.) prior to the peak of clinical disease. In both groups clinical disease peaked at ∼ day 14 p.i., but severity was increased from a score of 3.1 in wt mice to 4.0 in the GFAPγR1Δ group (Fig. 1A; Table 1).

**Table 1:** EAE in GFAPγR1Δ tg mice.

	wt C57BL/6	GFAPγR1Δ
Incidence	100% (n = 35)	100% (n = 40)
Mean day of onset	9±2	9±2
Peak Disease Score^a^	3.1±0.2	4.0±0.3*
Mortality^b^	3% (1/35)	38% (15/40)*

a Peak disease score determined at day 18 post immunization.

b Mortality determined at day 50 post immunization.

* p≤0.05.

**Figure 2:**
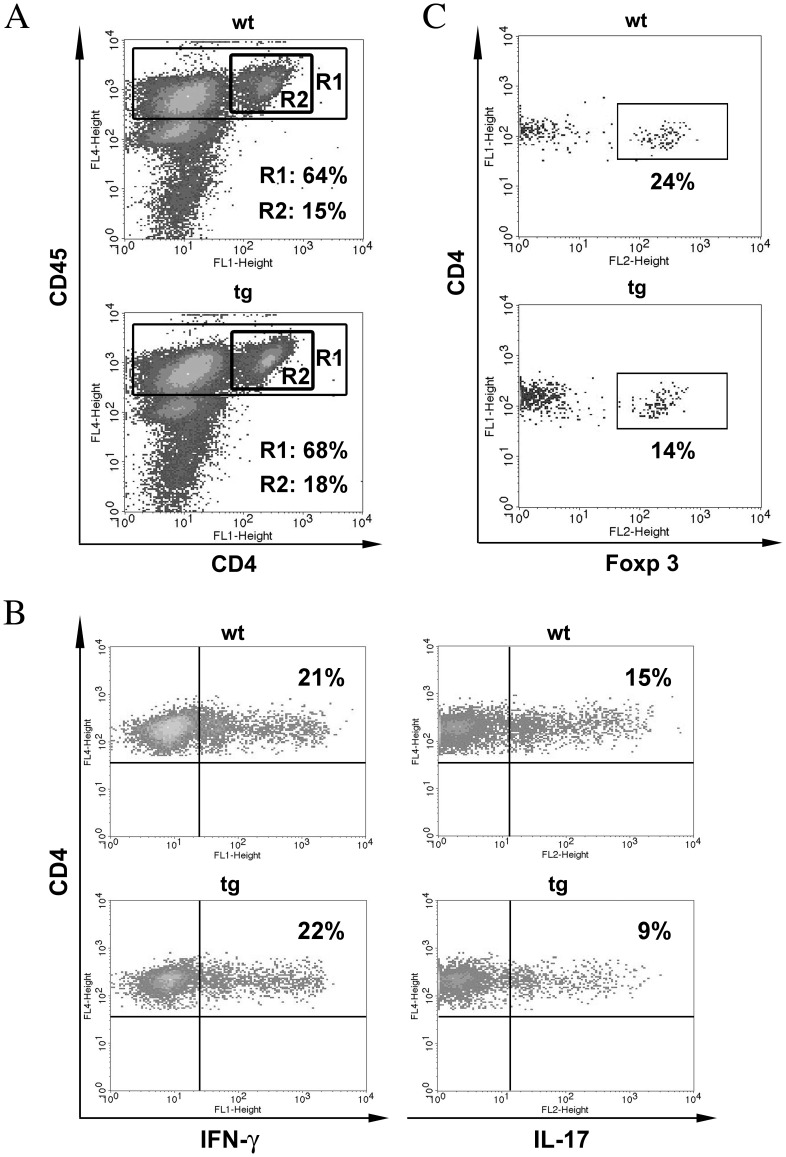
IFN-γ signaling to astrocytes does not influence brain inflammation. (**A**) CD45^hi^ bone marrow derived inflammatory cells and CD4^+^ T cells in brains (day 16 p.i.) analyzed by flow cytometry following isolation by Percoll gradients. R1 gate = CD45^hi^ inflammatory cells; R2 gate = CD4^+^ T cells within R1. Representative of 4 separate experiments at days 14 – 16 p.i. (**B**) IFN-γ and IL-17 secreting MOG-specific CD4^+^ T cells during acute EAE. Brain derived cells enriched by Percoll gradients at day 16 p.i. stimulated with MOG^35–55^ peptide for 4 hrs in the presence of GolgiStop. p>0.05 comparing the frequency of MOG-specific CD4^+^ T cells secreting IFN-γ; p<0.05 comparing IL-17 in wt and GFAPγR1Δ mice. CNS cells pooled from 4 – 5 mice per experiment. Data are representative of 3 – 4 separate experiments at days 14 – 16 p.i. (**C**) Foxp3^+^ regulatory T cells within CD4^+^ T cells enriched from brain at day 16 p.i. CNS cells pooled from 4 – 5 mice per experiment. p<0.05 comparing wt and GFAPγR1Δ mice. Data are representative of 3 separate experiments at days 14 – 16 p.i.

**Figure 3:**
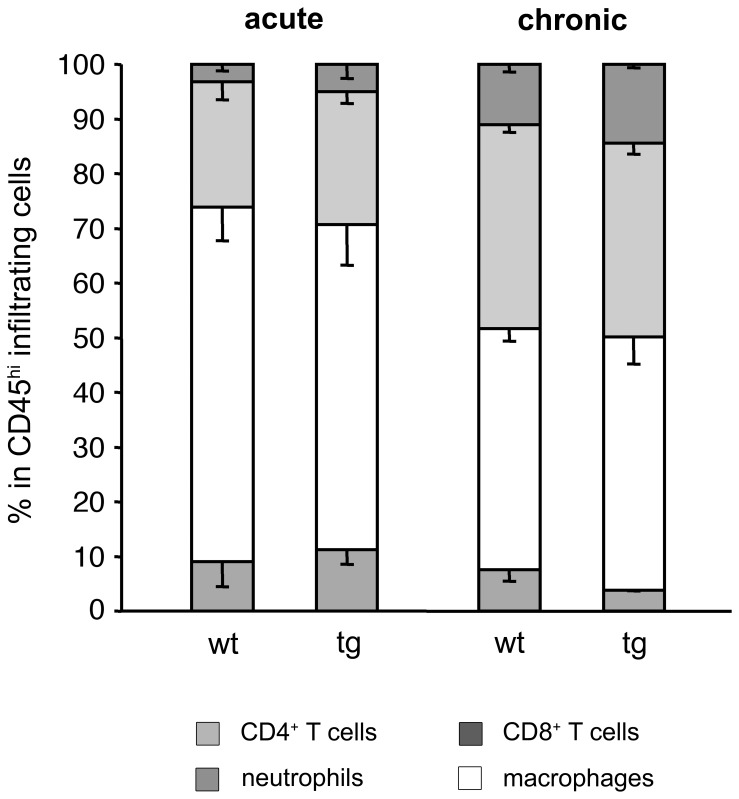
CNS inflammatory cell composition during acute and chronic EAE. Percentages of CD8^+^ T cells, CD4^+^ T cells, macrophages and neutrophils within the CD45^hi^ populations derived from spinal cords of mice during acute (day 16 p.i.) and chronic EAE (day 45 p.i.). Data derived from pools of 5 – 7 mice during acute EAE and 6 – 8 mice during chronic EAE. Average of ≥3 experiments per time point.

GFAPγR1Δ and wt mice were compared at the peak of acute disease to determine if IFN-γ signaling altered astrocyte activation or CNS inflammation. Astrocyte hypertrophy and GFAP expression (Fig. 1B) were similar in both groups, indicating no overt effects of IFN-γ on initial astrocyte reactivity. Furthermore, neither the extent of inflammation nor the anatomic distribution of inflammatory cells was altered (Fig. 1B). Flow cytometry confirmed no difference in the overall extent of CD45^hi^ inflammatory cells recruited into the CNS (Fig. 2A). Furthermore, percentages of CD4^+^ T cells (Fig. 2A), CD8^+^ T cells and macrophages within the infiltrates were also similar (Fig. 3). In contrast to the association between increased EAE severity and neutrophil accumulation in the global absence of IFN-γ [14], [15], only a small percentage of neutrophils were identified in the CNS of both groups by flow cytometry (Fig. 3); their low presence was confirmed by the inability to identify cells with the characteristic morphology of neutrophils in the brain by histopathology (Fig. 1B). The absence of increased neutrophils in the CNS of GFAPγR1Δ mice during acute disease suggested that clinical disease was aggravated by a mechanism distinct from global IFN-γ deficiency. In addition to the similar frequency of CD4^+^ T cells in the brains of the two groups (Fig. 2A), myelin oligodendrocyte glycoprotein (MOG) reactive CD4^+^ T cells secreting IFN-γ were also similar (Fig. 2B). By contrast, MOG specific CD4^+^ T cells secreting IL-17 (Fig. 2B) and Foxp3^+^ regulatory CD4^+^ T cells (Tregs) were decreased in GFAPγR1Δ mice compared to wt mice (Fig. 2C). Reduced Th17 and Tregs may be attributed to increased IFN-γ [10], [16]. Alternatively, as IFN-γ is protective in EAE [12]–[15], increased disease severity may reflect reduced bioavailable IFN-γ due to sequestration of IFN-γ binding to the dominant negative receptor. However, measurement of IFN-γ in cell free supernatants derived from dissociated brains at the peak of acute disease demonstrated protein levels of 2.1±0.2 ng/brain in wt mice versus 3.6±0.5 ng/brain in GFAPγR1Δ mice (n = 3; p<0.05). As overall frequencies of MOG reactive CD4^+^ T cells secreting IFN-γ were similar, increased IFN-γ in the brains of GFAPγR1Δ mice suggested enhanced secretion at the cellular level. Although a contribution of NK or CD8 T cells could not be excluded, these potential sources of IFN-γ were unlikely due to their low frequencies (NK ∼5% and CD8^+^ T cells ∼ 5%, see Fig. 3) and their equivalent frequencies in the wt and GFAPγR1Δ mice. Furthermore, reduced Th17 cell and Treg frequencies were consistent with suppression of these populations due to elevated IFN-γ [10], [16], [20].

**Figure 4:**
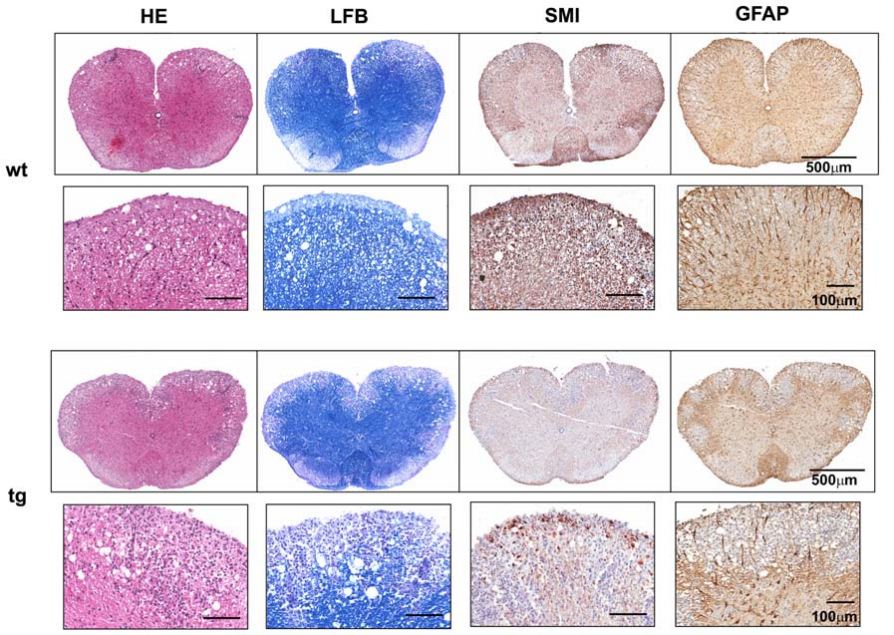
Astrocyte regulation of spinal cord demyelination and axonal loss during acute EAE. Cross section of spinal cord from wt (upper panels) and GFAPγR1Δ mice (lower panels) during acute disease (day 16 p.i.). Inflammation (HE), demyelination (LFB), axonal damage and loss (SMI), GFAP expression and hypertrophy of astrocytes in wt and GFAPγR1Δ mice. Data are representative of 3 separate experiments with 3 – 4 individuals per experiment and 6 cross sections per cord.

**Figure 5:**
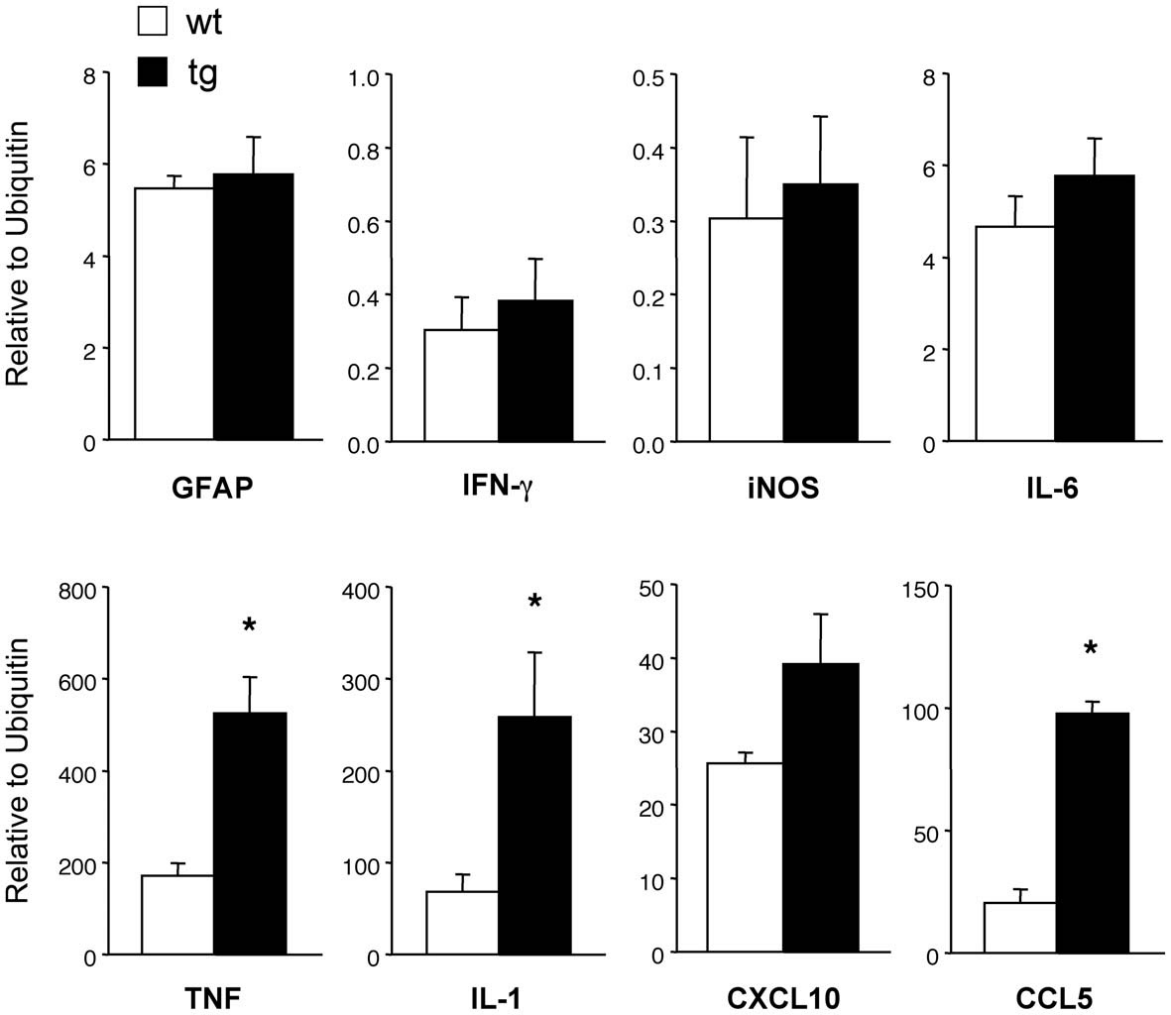
Astrocytes regulate IL-1, CCL5 and TNF mRNA during acute EAE. Relative gene expression in spinal cord from GFAPγR1Δ (average clinical score = 3.9) and wt mice (average clinical score = 3.1) during acute disease (day 18 p.i.) determined by qRT-PCR. Representative of 2 experiments (n = 3 – 4 mice/group) analyzed in triplicate. *p<0.05 comparing wt and GFAPγR1Δ mice.

Inflammation in spinal cords from the GFAPγR1Δ mice was also similar to wt controls at the peak of clinical symptoms (Fig. 4). Although numbers and distribution of CD4^+^ T cells were also similar (4.6±1.3/mm^2^ in GFAPγR1Δ mice vs. 3.8±0.1/mm^2^ in wt mice; Fig. S1), spinal cords of GFAPγR1Δ mice exhibited a ∼2-fold increase in demyelination (Fig. 4). Areas of demyelination encompassed 7.3±1.0% of spinal cord white matter in GFAPγR1Δ mice versus 3.8±0.9% in wt mice (p≤0.01). Furthermore, the increase in demyelination was associated with a prominent loss of axons in GFAPγR1Δ mice compared to controls (Fig. 4). Despite elevated demyelination and axonal loss in the absence of IFN-γ signaling to astrocytes, spinal cords showed no evidence of differential astrocyte activation by either immunohistochemistry (Fig. 4), or differences in GFAP mRNA expression during the peak of acute disease (Fig. 5). Although CCL5, IL-1 and TNF mRNA expression were increased in the spinal cords of the GFAPγR1Δ mice, no differences in IFN-γ, iNOS, CXCL10 or IL-6 mRNA were consistent with similar inflammation (Fig. 5). These data suggest that the initial disease pathogenesis, reflected by an increased demyelination in spinal cords, but not brain, during ascending paralysis is dampened by IFN-γ signaling to astrocytes.

### IFN-γ Signaling to Astrocytes Reduces Chronic Autoimmune Disease

Subsequent to peak disease severity the clinical symptoms in wt mice began a modest decline by day 16 p.i. (Fig. 1A). By contrast, GFAPγR1Δ mice not only exhibited increased severity of clinical symptoms following day 14 p.i., but the continued disease escalation was associated with increased mortality (Fig. 1A; Table 1). Sustained morbidity and significant mortality in GFAPγR1Δ mice after day 30 p.i. implied a critical role for IFN-γ induced astrocyte signaling in neuroprotection and limiting disability. A similar absence of clinical remission was found in a preliminary experiment comparing EAE SJL mice carrying the GFAPγR1Δ gene (data not shown). These data suggest that astrocyte responses to IFN-γ are protective, irrespective of genetic background.

**Figure 6:**
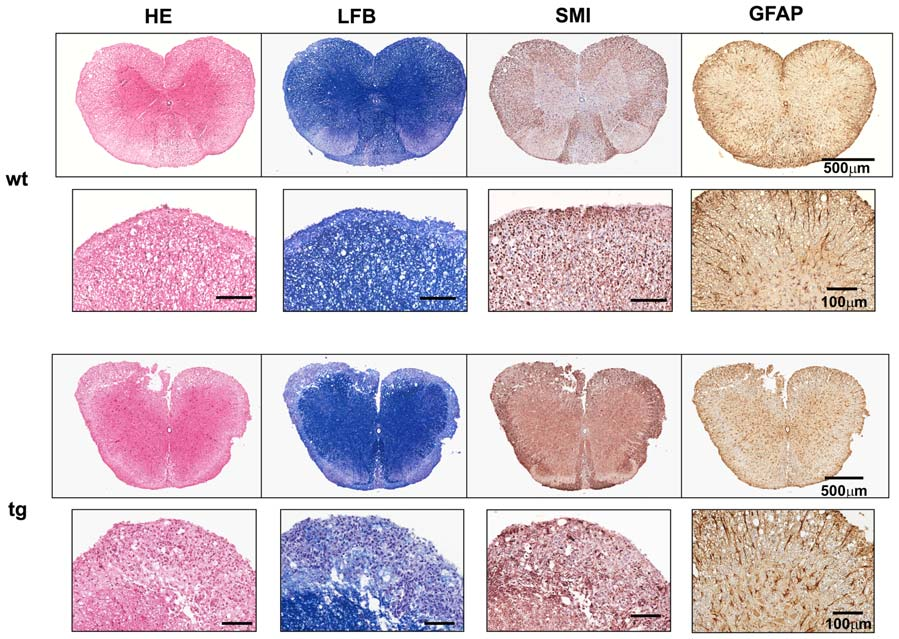
Sustained demyelination, axonal loss and astrocyte activation during chronic disease. Cross section of spinal cord from wt (upper panels) and GFAPγR1Δ mice (lower panels) during chronic disease (day 35 p.i.). Inflammation (HE), demyelination (LFB), axonal damage and loss following visualization with mAb SMI31 and SMI32 (SMI), GFAP expression and hypertrophy of astrocytes (GFAP) in wt and GFAPγR1Δ mice. Data are representative of 3 separate experiments with 3 – 4 individuals per experiment and 6 cross sections per spinal cord.

**Figure 7:**
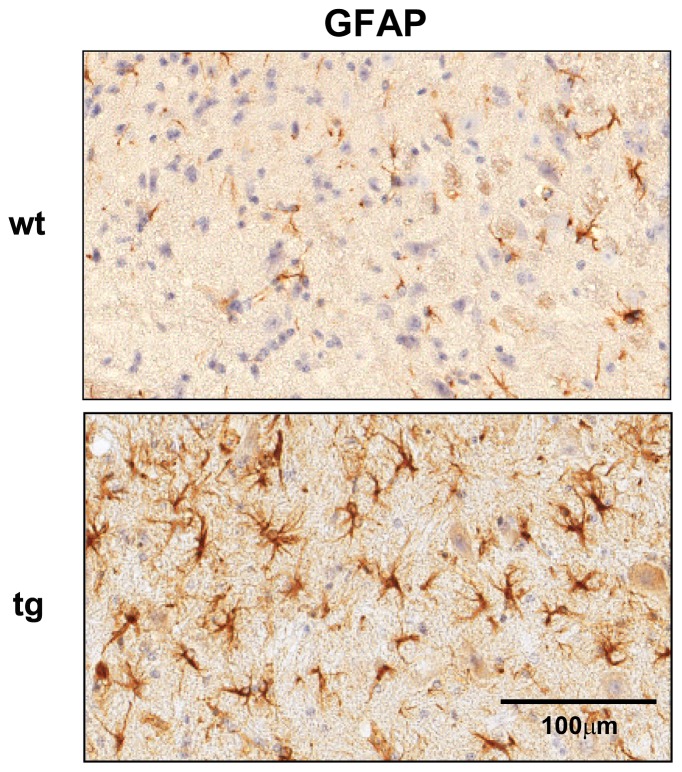
IFN-γ signaling regulates astrocyte activation within grey matter during chronic EAE. Cross section of spinal cord grey matter from wt (upper panel) and GFAPγR1Δ mice (lower panel) during chronic disease (day 35 p.i.). Grey matter regions shown are distinct from areas of demyelination. Data are representative of 3 separate experiments with 3 – 4 individuals per experiment and 6 cross sections per spinal cord and 2 separate experiments in which the spinal cords were sectioned longitudinally.

Escalating disease in GFAPγR1Δ mice coincided with focal areas of intense inflammation in spinal cords, which contrasted with the more diffuse inflammation in wt mice (Fig. 6). Demyelination was also increased with myelin loss encompassing 5.7±1.8% of spinal cord area in GFAPγR1Δ mice versus 2.3±1.4% in wt mice (p≤0.05) at day 35 p.i. (Fig. 6). The demyelinated areas further exhibited enhanced axonal damage in GFAPγR1Δ mice (Fig. 6), supporting a correlation between enhanced tissue damage, sustained morbidity and increased mortality (Fig. 1A; Table 1). Astrocyte activation associated with areas of myelin loss is a prominent finding in the CNS of both patients with MS and rodents with EAE. Although demyelination was increased in the CNS of GFAPγR1Δ mice, the extent of astrocyte activation associated with spinal cord white matter lesions was similar in both groups (Fig. 6; ∼60 GFAP^+^ cells/mm^2^). By contrast, the frequency of activated astrocytes in spinal cord grey matter areas that were not associated with demyelinated lesions, was increased in GFAPγR1Δ mice with 101.5±23.0 GFAP^+^ activated astrocytes/mm^2^ versus 25.5±15.5 in wt mice (p<0.001) (Fig. 7). A similar increase in astrocyte activation within grey matter distal to white matter lesions was also detected in GFAPγR1Δ SJL mice during chronic EAE (data not shown).

### IFN-γ Dependent Astrocyte Regulation of Inflammation

**Figure 8:**
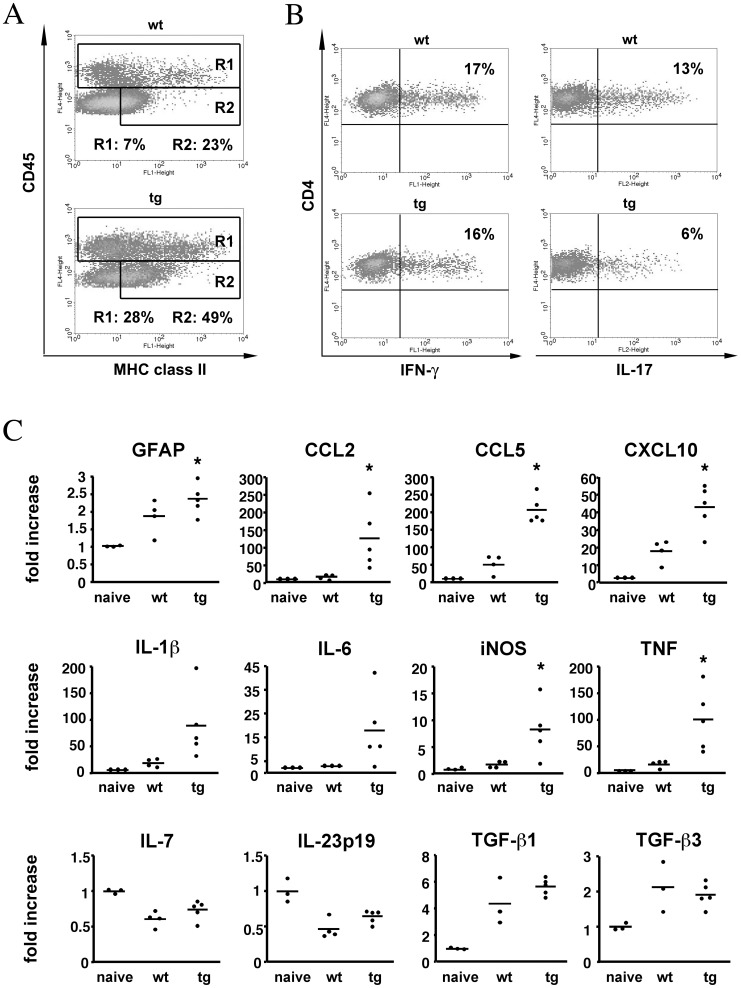
Sustained inflammation and increased pro-inflammatory genes during chronic EAE. (**A**) CD45^hi^ inflammatory cells and MHC class II expression on CD45^low^ microglia from spinal cord during chronic disease (day 42 p.i.). R1 gates depict total inflammatory cells. The R2 gate depicts relative MHC Class II expression. Representative of 4 similar experiments. (**B**) Relative frequencies of MOG specific IFN-γ and IL-17 CD4^+^ T cells derived from spinal cord during chronic disease (day 42 p.i.). Pools of 5 – 7 mice per experiment. Representative of 2 separate experiments. (**C**) Relative gene expression in spinal cord mRNA from GFAPγR1Δ (average clinical score = 3.9) and wt mice (average clinical score = 0.9) during chronic disease (day 43 p.i.) determined by qRT-PCR. Representative of 2 experiments (n = 3 – 4 mice/group) analyzed in triplicate. *p<0.05 comparing wt and GFAPγR1Δ mice.

Flow cytometric analysis during chronic disease revealed an ∼4-fold increase in CD45^hi^ inflammatory cells confirming increased inflammation in the absence of IFN-γ signaling to astrocytes (Fig. 8A). Similar to the acute disease, relative percentages of neutrophils, macrophages, CD4^+^ and CD8^+^ T cells were all similar (Fig. 3). Furthermore, no differences in expression of activation markers on CNS derived CD4^+^ T cells, including CD69, CD122, CD127, Fas, FasL, ICOS and CD152 were evident between the groups (data not shown). The percentages of MOG reactive Th1 cells were also not altered in the CNS of GFAPγR1Δ relative to wt mice (Fig. 8B) and the decreased percentage of potentially destructive Th17 cells identified during acute disease (Fig 2), was also sustained during chronic disease (Fig. 8B). Equivalent expression of IL-7, IL-23 and TGF-β mRNA (Fig. 8C) suggested that the decrease in Th17 cells was dependent upon an increase in IFN-γ and not related to a defect in activation or maintenance signals [20]. Increased inflammation and sustained MHC class II expression on microglia (Fig. 8A) further suggested that IFN-γ signaling to astrocytes down regulates inflammation universally without altering the composition of the CNS infiltrates. This concept is supported by sustained composition of CNS infiltrates during inflammation induced astrocyte apoptosis [21].

**Figure 9:**
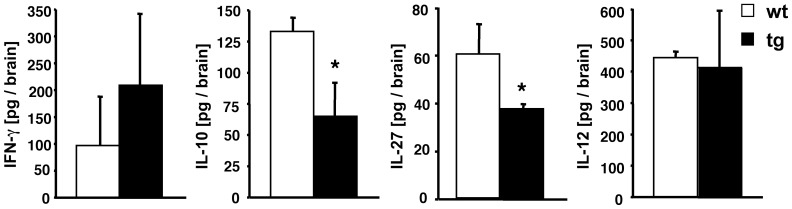
IFN-γ signaling to astrocytes altered cytokine expression. Comparative IFN-γ, IL-27, IL-10 and IL-12 levels in brains derived from GFAPγR1Δ (average clinical score = 4.2) and wt mice (average clinical score = 1.8) during chronic disease (day 42 p.i.). Cytokine levels over naïve controls determined in tissue homogenate by ELISA. *p<0.05 comparing wt and GFAPγR1Δ mice.

**Figure 10:**
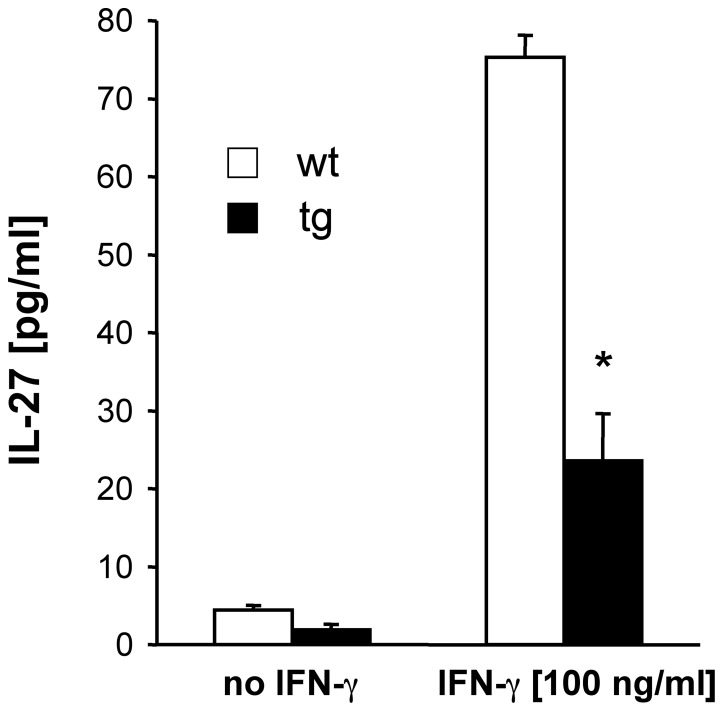
IFN-γ dependent secretion of IL-27 by astrocytes. IL-27 secretion by astrocytes derived from wt and GFAPγR1Δ tg mice. Cultures were treated with 100 ng/ml rIFN-γ for 48 hr. IL-27 concentrations determined by ELISA. Representative of 3 experiments. *p<0.05 comparing wt and GFAPγR1Δ mice.

The inability to restrain ongoing inflammation during EAE in GFAPγR1Δ mice was evident at multiple levels. Astrocyte activation was sustained consistent with increased expression of GFAP mRNA. CCL2, CCL5 and CXCL10 mRNA levels were increased (Fig. 8C). The expression of mRNA encoding potentially destructive immune mediators including IL-1, IL-6, iNOS, and TNF were all increased (Fig. 8C). IFN-γ was ∼2-fold higher in the CNS of GFAPγR1Δ mice (Fig. 9). Lastly, the level of the anti-inflammatory cytokine IL-10 was reduced (Fig. 9), suggesting limited availability of IL-10 may be a key in prolonging astrocyte activation and inflammation [22]. A possible link between reduced IL-10 and the inability of IFN-γ signaling to astrocytes is provided by the capacity of IFN-γ to induce IL-27 in astrocytes [23], thereby promoting IL-10 production. Indeed IL-27 in the CNS of the GFAPγR1Δ mice was significantly reduced compared to wt mice during chronic disease (Fig. 9). By contrast, IL-12 (Fig. 9), an indirect suppressor of CNS inflammation by promoting IFN-γ production [24], was similar in the CNS of both GFAPγR1Δ and wt mice. Astrocytes derived from GFAPγR1Δ and wt mice were stimulated with IFN-γ to confirm IFN-γ dependent IL-27 secretion by astrocytes [25]. While IFN-γ induced IL-27 secretion by astrocytes from wt mice, IL-27 secretion was significantly reduced in cultures derived from GFAPγR1Δ mice (Fig. 10). These data support the possibility that IFN-γ mediated IL-27 secretion by astrocytes regulates EAE effector T cell function, inflammation and tissue destruction via induction of IL-10; however, this does not exclude the possibility that the inability of astrocytes to respond to IFN-γ could directly or indirectly dysregulate a variety of other immunomodulatory functions [4], [5], [17].

## Discussion

In the EAE model of MS, IFN-γ functions as a pro-inflammatory cytokine in the early stages of disease [10], [11], [26], yet it also assumes a prominent anti-inflammatory role during the transition to remission [10], [12]–[15]. Protection has been attributed to a variety of potentially interrelated mechanisms including limiting neutrophil accumulation, Th17 cell activation, IL-1R signaling, matrix metalloproteinase secretion, pro-inflammatory cytokine secretion and chemokine activity; in addition IFN-γ facilitates T cell apoptosis and protects oligodendrocytes from death via an ER stress response [10], [11], [17]. The activities of astrocytes during autoimmunity also range from pro- to anti-inflammatory [3]–[5]. However, to what extent a direct action of IFN-γ on astrocytes contributes to inhibitory mechanism is unclear. The data herein demonstrate that among the multiple cells in the CNS expressing the IFN-γ receptor, the astrocyte is the prominent cellular target of IFN-γ mediated anti-inflammatory activity, thus promoting remission.

Early functions of astrocytes imposed by innate responses are largely pro-inflammatory during EAE [3], [6], [7], [26]. For example, astrocytes contribute to the loss of BBB integrity via secretion of reactive oxygen species, chemokines and pro-inflammatory cytokines [3]–[5]. This pro-inflammatory milieu is associated with initial axonal damage prior to accumulation of self reactive T cells [27], which further promotes immune mediated damage. Blocking the pro-inflammatory transcription factor NF-κB in astrocytes improves recovery during chronic EAE [28], [29]. Supporting an initial pro-inflammatory role of astrocytes dependent on innate, not IFN-γ responsiveness, inhibition of IFN-γ signaling to astrocytes did not influence EAE onset or incidence, initial disease progression, astrocyte activation, or BBB integrity as indicated by similar entry of inflammatory cells into the brain. By contrast, an inflammation dampening effect of IFN-γ became evident during the onset of disease remission. Astrocytes limit ongoing inflammation and pathogenic processes at several levels. They not only facilitate repair by inhibiting inflammatory cell entry into the CNS parenchyma [6], [7], [30], but also down regulate T cell effector function and proliferation [4], [5], [21]. For example, EAE in GFAP deficient mice results in more severe and widespread inflammation [31]. In addition, T cell-astrocyte interactions as well as astrocyte secretion of an unidentified soluble product, distinct from nitric oxide, prostaglandins, or tryptophan metabolism, suppress T cell proliferation [4], [5]. T cell proliferation was also not inhibited via defective IL-2 release, despite the suggestion that T cell-astrocyte interactions facilitate secretion of anti-inflammatory cytokines [4], [5]. Lastly, astrocytes may limit inflammation by triggering apoptosis in T cells [32]. Nevertheless, the role of IFN-γ signaling in these potentially anti-inflammatory, protective mechanisms is not clear.

Elevated IFN-γ in the CNS of GFAPγR1Δ mice during both acute and chronic disease excluded limited IFN-γ due to sequestration by the transgenic receptor as a mediator of increased clinical severity and mortality. Indeed, enhanced IFN-γ production may reflect an attempt to compensate for the loss of IFN-γ dependent astrocyte mediated control of inflammation [33]. Sustained expression of pro-inflammatory cytokines, particularly IL-6 and TNF, thus represents a primary mechanism underlying ongoing tissue destruction in GFAPγR1Δ mice. IL-6 is known to mediate neurological dysfunction [34] and astrocytes are the predominant source of IL-6 during CNS autoimmune disease. Furthermore, its secretion is down regulated by IFN-γ induced SOCS activity [17]. On the other hand, sustained TNF implicated dysregulated activation of microglia or macrophages via increased IFN-γ or IL-6, as TNF is predominantly secreted by activated CNS macrophages and microglia during EAE [35], [36]. However, the down regulation of microglia activation, including TNF secretion, following interaction with activated astrocytes [37] questions this notion. While both IL-6 and TNF may thus contribute to sustained pathological changes, the source of TNF remains unclear. Similarly, astrocytes are a potential source of the IFN-γ induced chemokine CXCL10 during EAE, one of the chemokines controlling T cell recruitment [38]. However, the increased expression of CXCL10 coupled with the inability of the astrocytes in the GFAPγR1Δ mice to respond to IFN-γ suggests altered microglia activation and secretion of CXCL10 [38] or activation of CXCL10 transcription via an independent signaling pathway mediated by TNF or Type 1 interferons [39], [40].

Importantly, pathology further correlated with decreases in both Tregs and IL-10, but not with increased antigen specific Th17 cells, although astrocytes are critical targets of IL-17 [41]. Although it is possible that the increased IFN-γ in the CNS influenced peripheral activation of antigen specific Th17 cells, previous data demonstrated no evidence for expression of the transgene in peripheral organs, including lymphoid organs [18]. IL-10, secreted by a variety of cells types including CD4^+^ T cells, CD8^+^ T cells and Tregs [42], inhibits both acute and chronic EAE [43], [44]. The decrease in this anti-inflammatory cytokine suggests that the induction of IL-27 secretion is a critical aspect of astrocyte responses to IFN-γ, thus promoting IL-10 secretion by T cells [23], [25]. However, our data is unable to distinguish the contribution of paracrine versus autocrine IFN-γ induced signals to astrocytes on IL-10 production, as IL-10 also regulates astrocyte activation [22], and can itself be secreted by astrocytes to exert autologous functions [45].

In addition to mediating an imbalance of protective versus detrimental cytokines, our results demonstrate that IFN-γ signaling to astrocytes limits the extent of astrocyte activation during chronic EAE, specifically in grey matter. Activated astrocytes, especially within and adjacent to areas of demyelination are a prominent feature of white matter plaques associated with both chronic MS and EAE [3]–[5]. Astrocytes protect neurons and oligodendroglia, but also form glial scars which inhibit regeneration after neuronal injury [3], [5], [8], [20]. The absence of reactive astrocytes increases axonal regeneration after injury [46], consistent with the concept that limiting astrogliosis is critical for axonal regeneration after neuronal injury. While the significance of sustained astrocyte activation in grey matter tracks is unclear in our model, it is consistent with increased axonal damage, and suggests possible damage to axons outside the lesions, which may not be manifested by histological analysis.

Limiting inflammation following either infection or during an autoimmune attack is a prerequisite for the initiation of repair. This is critically important within the CNS which has limited regenerative capacity. In summary, our data identify astrocytes as prominent targets underlying IFN-γ mediated suppression of chronic CNS inflammation [19]. The data further provide a link between sustained inflammatory responses, enhanced demyelination and axonal degeneration associated with loss of neurological function during EAE and chronic progressive MS. Although the inability of astrocytes to respond to IFN-γ did not alter disease in the brain, engagement of the IFN-γ receptor on astrocytes in spinal cord limits demyelination and functions in a neuroprotective capacity. Current therapies for MS are primarily focused on anti-inflammatory and immunomodulatory approaches and have been partially successful in treating acute episodes. The identification of astrocytes as critical responders and mediators of IFN-γ signaling in limiting CNS autoimmune disease may provide insights into new approaches to limit long term progression to disability.

## Materials and Methods

### Mice

Homozygous H-2^b^ GFAP/IFN-γR1ΔIC (GFAPγR1Δ) transgenic mice [18], expressing a dominant negative IFN-γ receptor alpha chain under control of GFAP promoter, were bred locally. C57BL/6 (H-2^b^) wild type (wt) mice were purchased from the National Cancer Institute (Frederick, MD). All animal experiments were carried out in accordance with the National Institutes of Health Guide for the Care and Use of Laboratory Animals. Procedures were performed in compliance with protocols approved by the Cleveland Clinic Institutional Animal Care and Use Committee (protocol number: 2011-0439) and all efforts were made to minimize suffering.

### EAE

EAE was induced by immunization with myelin oligodendrocyte glycoprotein (MOG)^35–55^ peptide emulsified at 3 mg/ml in PBS with an equal amount of incomplete Freund’s adjuvant (IFA; Sigma-Aldrich, St. Louis, MO) supplemented with 5 mg/ml *Mycobacterium tuberculosis*, strain H37Ra (Difco, Detroit, MI). Mice were immunized subcutaneously with 200 µl emulsion distributed over two sites on the flanks. Pertussis toxin (Sigma-Aldrich), 200 ng in 200 µl PBS, was injected intraperitoneally on the day of initial immunization. Mice received a subcutaneous booster MOG^35–55^ immunization on day 7. Animals were scored for clinical symptoms as follows: 0 = no signs of disease; 1 = flaccid tail or hind limb weakness; 2 = flaccid tail and hind limb weakness, loss of righting reflex; 3 = partial hind limb paralysis; 4 = complete hind limb paralysis; 5 = moribund or dead.

### Flow Cytometry

Brains and spinal cords from perfused mice were homogenized separately as previously described [47], [48]. Homogenates were centrifuged at 450×g for 7 min at 4°C. Supernatants were stored at -80°C for cytokine determination (see below). Cells were resuspended in 30% Percoll (Amersham Biosciences, Piscataway, NJ) and isolated by centrifugation (800×g for 30 min at 4°C) onto 70% Percoll cushions. Non-specific binding was inhibited by incubation with anti-CD16/CD32 (2.4G2; BD Biosciences, San Diego, CA) and a 10% mixture of normal goat, human, mouse and rat serum for 10 min on ice. FITC, PE, PerCP, and APC conjugates with monoclonal antibodies (mAb) used to identify and quantify microglia and inflammatory cells were: CD4 (GK1.5), CD8a (53-6.7), CD45 (30-F11), MHC class II (2G9), Ly6G (1A8) (BD Biosciences), and F4/80 (Serotec, Raleigh, NC). Microglia and inflammatory cells were distinguished based on differential CD45 expression. CD4^+^ T cells were identified as CD45^hi^CD4^+^, CD8^+^ T cells as CD45^hi^CD8^+^, macrophages as CD45^hi^F4/80^+^ and neutrophils as CD45^hi^Ly6G^+^MHC class II^-^. MOG^35–55^ specific induction of cytokines was determined by stimulation of 1×10^6^ CNS cells with 20 µg/ml peptide for 6 h at 37°C with GolgiStop (BD Biosciences) added for the last 4 h. Intracellular cytokines were detected with FITC-anti-IFN-γ (clone XMG1.2; BD Biosciences) and PE-anti-IL-17 (clone TC11-18H10; BD Biosciences). Intracellular Foxp3 was detected by staining for cell surface markers, followed by permeabilization with Fixation/Permeabilization Reagent (eBioscience, San Diego, CA) and incubation with PE-labeled anti-Foxp3 (FJK-16s; eBioscience). Cells were analyzed on a FACSCalibur flow cytometer (BD Biosciences) using CellQuest Pro software (BD Biosciences). Data was analyzed using FlowJo (7.6.1) software (Tree Star Inc., Ashland, OR).

### Cytokine Determinations

Cytokines were determined by ELISA using antibody pairs and recombinant cytokine standards from BD Bioscience. IL-27 was measured using QUANTIKINE Mouse IL-27p28 Immunoassays (R&D Systems Inc., Minneapolis, MN).

### Histopathology

Following anesthesia, mice were perfused with PBS (pH 7.2). Brains and spinal cords were fixed with Clark’s solution (75% ethanol and 25% glacial acetic acid), and embedded in paraffin. Spinal cords were divided into 6 sections prior to embedding, corresponding to cervical, thoracic and lumbar levels. Cross sections (6 µm), were stained with either hematoxylin and eosin (H&E) or luxol fast blue (LFB). Immunoperoxidase staining was used to identify activated astrocytes with anti-GFAP (AbCam, Cambridge, MA) and axonal integrity with SMI-31 and SMI-32 mAb (Sternberger Monoclonals Inc., Lutherville, MD) followed by visualization using Vectastain ABC kit (Vector Laboratories, Burlingame, CA) and 3,3′-diaminobenzidine (Sigma-Aldrich). Sections from at least 3 separate experiments containing at least 3 mice per group were reviewed in a blinded manner. Numbers of GFAP^+^ cells in spinal cord were determined in 15 non-overlapping 40× fields (0.2 mm^2^) in white matter and gray matter. Stained spinal cord sections of all 6 levels on individual glass slides were scanned (40×) and digitally imaged at high resolution with an Aperio ScanScope (Vista, CA). Aperio software was used to quantify areas of demyelination within the white matter tracks of each of the 6 sections per individual mouse. For analysis of CD4^+^ T cells spinal cords were embedded in Tissue-Tek O.C.T. (Andwin Scientific, Tryon, NC), flash frozen in liquid nitrogen and stored at −80°C. Blocks were warmed to −20°C prior to cutting 10 µm sections by cryostat. Following fixation with acetone for 2 min at 4°C, non-specific antibody binding was blocked with Cyto Q Background Buster (Innovex Biosciences, Richmond, CA) for 15 min. Sections were stained with anti-CD4 (L3T4) antibody (Vector Laboratories) diluted in Cyto Q Immuno Diluent (Innovex Biosciences) followed by biotinylated rabbit anti-rat, peroxidase ABC reagent and visualized with NovaRED substrate (all from Vector Laboratories). Sections were counter stained with hematoxylin to visualize the nuclei. Spinal cord sections were scanned (40×) and digitally imaged at high resolution with an Aperio ScanScope.

### Astrocyte Cultures

Mixed glial cultures (∼70% astrocytes) were established from neonatal GFAPγR1Δ and wt mice as previously described [47]. IL-27 secretion was determined 48 h after rIFN-γ (100 ng/ml) stimulation.

### Gene Expression Analysis

Frozen tissues were homogenized in TRIzol (Invitrogen, Carlsbad, CA) and cDNA prepared as described [47], [48]. Quantitative real-time PCR was performed using 4 µl of cDNA and SYBR Green Master Mix (Applied Biosystems, Foster City, CA) in duplicate on a 7500 Fast Real-Time PCR System (Applied Biosystems). Expression levels were normalized to ubiquitin or GAPDH using the following formula: (2^[CT {GAPDH} - CT {Target}]^) x 1000, where CT is the threshold cycle. Samples from individual mice were analyzed in duplicate or triplicate with 3 or more mice per group.

### Statistical Analysis

Statistical significance was determined by two-tailed Student’s *t* test. A value of p<0.05 was considered statistically significant.

## Supporting Information

Figure S1 **CD4^+^ T cell recruitment into the spinal cord during acute EAE.** CD4^+^ T cells were visualized in 10 µm frozen sections of spinal cords from wt and GFAPγR1Δ tg mice at day 18 p.i. Immunoperoxidase stain (NovaRED chromogen with hematoxylin counterstain). Scale bars = 50 microns.
(TIF)
