# Supplementary figures and images for: IFN-γ Signaling to Astrocytes Protects from Autoimmune Mediated Neurological Disability
Source: PLoS One. 2012 Jul 27;7(7):e42088. doi: 10.1371/journal.pone.0042088 (PMC3407093; doi:10.1371/journal.pone.0042088)

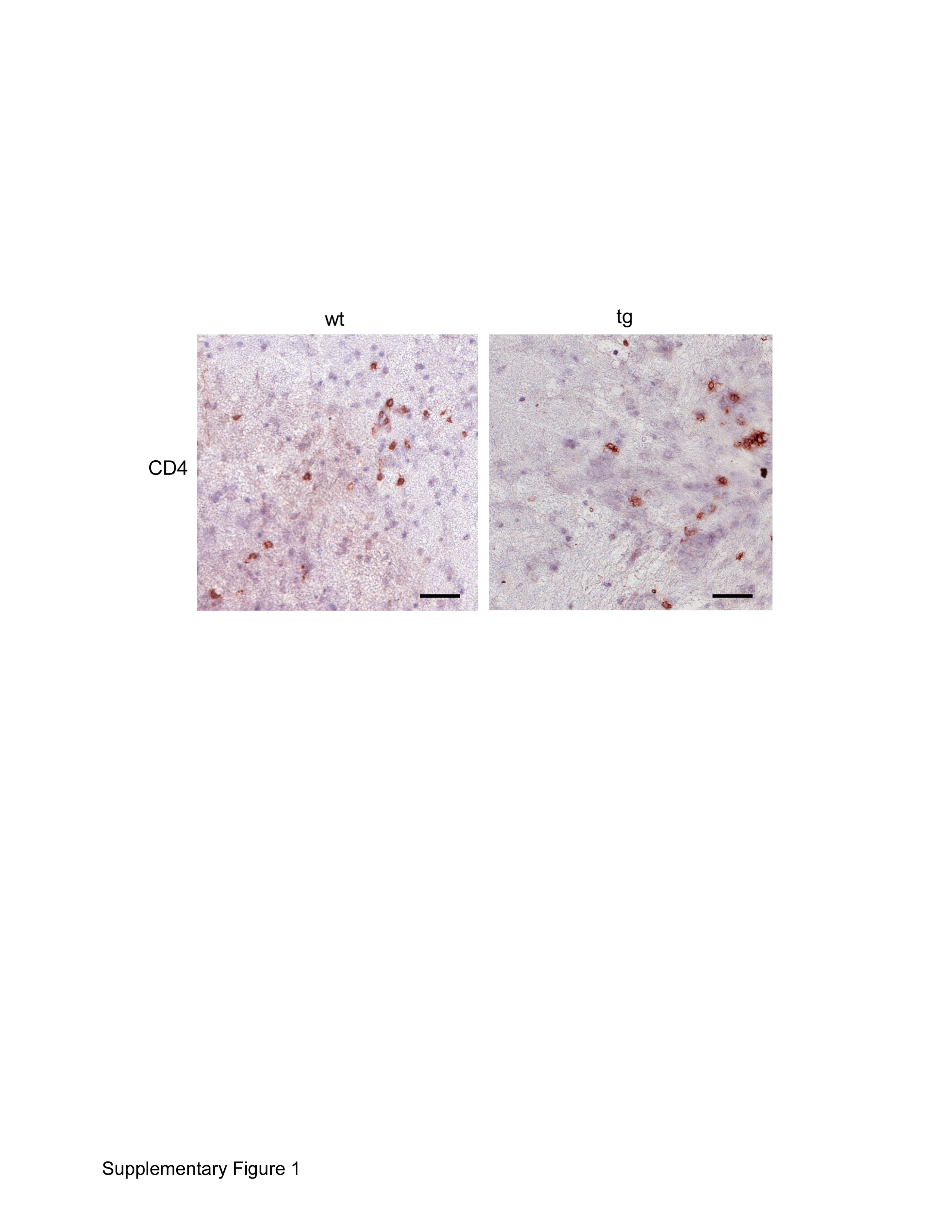

Supplement: Figure S1 — CD4+ T cell recruitment into the spinal cord during acute EAE. CD4+ T cells were visualized in 10 µm frozen sections of spinal cords from wt and GFAPγR1Δ tg mice at day 18 p.i. Immunoperoxidase stain (NovaRED chromogen with hematoxylin counterstain). Scale bars = 50 microns. (TIF) [file pone.0042088.s001.tif]
